# Disulfidptosis, A Novel Cell Death Pathway: Molecular Landscape and Therapeutic Implications

**DOI:** 10.14336/AD.2024.0083

**Published:** 2024-05-02

**Authors:** Qiuyang Gu, Yumei An, Mingyuan Xu, Xinqi Huang, Xueshi Chen, Xianzhe Li, Haiyan Shan, Mingyang Zhang

**Affiliations:** ^1^Institute of Forensic Sciences, Suzhou Medical College, Soochow University, Suzhou, China.; ^2^Department of Obstetrics and Gynecology, The Affiliated Suzhou Hospital of Nanjing Medical University, Suzhou, China

**Keywords:** cell death, disulfidptosis, ferroptosis, cuproptosis, pyroptosis, injury, cancer

## Abstract

Programmed cell death is pivotal for several physiological processes, including immune defense. Further, it has been implicated in the pathogenesis of developmental disorders and the onset of numerous diseases. Multiple modes of programmed cell death, including apoptosis, pyroptosis, necroptosis, and ferroptosis, have been identified, each with their own unique characteristics and biological implications. In February 2023, Liu Xiaoguang and his team discovered “disulfidptosis,” a novel pathway of programmed cell death. Their findings demonstrated that disulfidptosis is triggered in glucose-starved cells exhibiting high expression of a protein called SLC7A11. Furthermore, disulfidptosis is marked by a drastic imbalance in the NADPH/NADP+ ratio and the abnormal accumulation of disulfides like cystine. These changes ultimately lead to the destabilization of the F-actin network, causing cell death. Given that high SLC7A11 expression is a key feature of certain cancers, these findings indicate that disulfidptosis could serve as the basis of innovative anti-cancer therapies. Hence, this review delves into the discovery of disulfidptosis, its underlying molecular mechanisms and metabolic regulation, and its prospective applications in disease treatment.

## 1. Introduction

Programmed cell death, which is a highly regulated, orderly process of cell destruction, is orchestrated by a molecular cascade controlled by a series of proteins, enzymes, hormones, and cytokines [1, 2]. Multiple modes of programmed cell death have been discovered and explored in detail so far, including apoptosis, pyroptosis, necroptosis, and ferroptosis [3, 4]. Research into these types of programmed cell death is of great significance. This is because these processes are not only involved in innate immune defense against bacterial and viral infections [5], but they also contribute to developmental disorders and neurodegenerative diseases [3, 6, 7]. Various forms of mitochondria-related programmed-cell- death have also been implicated in certain diseases associated with aging, including neurodegenerative, cardiovascular, and metabolic diseases [8]. Avoiding these different types of cell death is crucial for cancer cells and allows them to proliferate and achieve metastasis. Accordingly, the induction of programmed cell death could be effective in controlling the survival and spread of malignant cells [9]. Hence, insights into programmed cell death could improve our understanding of disease pathogenesis and offer new therapeutic strategies for some diseases.

In February 2023, Liu Xiaoguang and colleagues reported their discovery of a novel type of programmed cell death, disulfidptosis [10]. They demonstrated that the process of disulfidptosis occurs in cells exhibiting high solute carrier family 7 member 11 (SLC7A11, a transport protein involved in a transport system for exchanging cystine and glutamate.) levels under conditions of glucose starvation and is induced by disulfide stress (a cellular condition marked by oxidative stress due to the excessive generation or accumulation of proteins with disulfide bonds). Disulfidptosis involves the depletion of nicotinamide adenine dinucleotide phosphate (NADPH) (i.e., an imbalance in the NADPH/NADP+ ratio) and the abnormal accumulation of disulfides like cystine. These changes eventually induce cell death by causing the collapse of the Filamentous actin (F-actin) network [11-13]. Thus, in cancers marked by a high expression of SLC7A11, disulfidptosis presents a therapeutic opportunity to control cancer progression via pharmacological or genetic interventions. In this review, we discuss how disulfidptosis was discovered and detail its known molecular mechanisms and metabolic regulation. In addition, we focus on the potential applications of disulfidptosis in cancer treatment and propose disulfidptosis-based anti-cancer strategies.

## 2. Overview of Disulfidptosis

**Figure 1.**
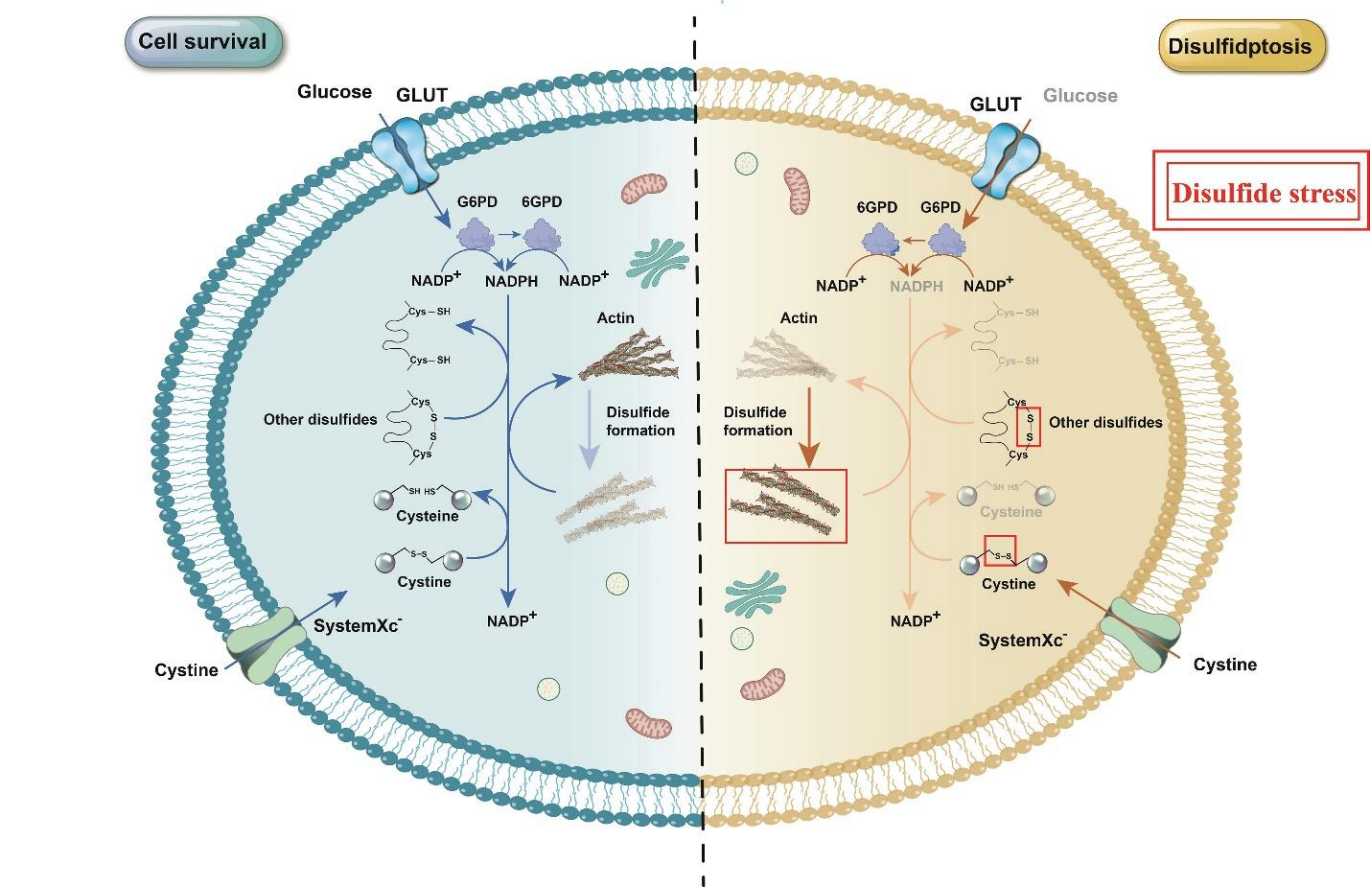
**Mechanism of disulfidptosis**. Under normal conditions, sufficient amounts of glucose are transported into the cell, and a large amount of NADPH is generated through the pentose phosphate pathway. This NADPH reduces the cystine transported by SLC7A11 into cysteine. Simultaneously, the intracellular reducing environment also prevents the formation of other disulfide bonds, thus protecting the cell from disulfide stress and ensuring cell survival. However, in cells undergoing disulfidptosis, conditions of glucose starvation or GLUT inhibition lead to reduced glucose uptake and diminished NADPH production, resulting in the collapse of the cellular redox environment. The consequent accumulation of intracellular cystine and other disulfides triggers disulfide stress. This leads to disulfide bond formation in actin filaments, collapse of the F-actin cytoskeleton, and ultimately induces cell death. Abbreviations: NADPH, nicotinamide adenine dinucleotide phosphate; G6PD, glucose-6-phosphate dehydrogenase; 6GPD, 6-phosphogluconate dehydrogenase; Cys, Cysteine.

The study conducted by Liu et al. focused on cells exhibiting two key features: (1) glucose starvation, and (2) high SLC7A11 expression. They observed that glucose starvation significantly reduces the amount of glucose taken up by cells, leading to a substantial decrease in NADPH synthesis through the pentose phosphate pathway (PPP). Simultaneously, high amounts of NADPH are also consumed due to extensive transport, resulting from the high expression of SCL7A11, and the reduction of intracellular disulfides. As a result, cells show a severe deficiency of NADPH, and their NADPH/NADP+ balance is disrupted. This destroys the cell’s redox balance. Additionally, the high expression of SCL7A11 also causes a significant intracellular accumulation of cystine. However, the excess cystine cannot be converted into cysteine due to the unavailability of NADPH. This leads to additional disulfide stress and cytotoxicity [10] (Fig. 1).

Initial studies showed that during glucose starvation, cells with high SLC7A11 expression undergo a unique form of cell death that is closely related to disulfide accumulation. At first, researchers attempted to understand whether ferroptosis inhibitors (Ferr-1 and DFO), apoptosis inhibitors (Z-VAD-fmk), necroptosis inhibitors (Nec-1 and Nec-2), and autophagy inhibitors (CQ) can prevent this form of cell death. However, these known cell death inhibitors could not attenuate the cell death induced by high SLC7A11 expression following glucose starvation. Similarly, the knockout of genes related to ferroptosis (*ACSL4*) and apoptosis (*BAX* and *BAK*) also did not affect this form of cell death, i.e., disulfidptosis. Interestingly, however, thiol oxidants (diamide and maleic acid diethyl ester) were found to significantly promote cell death in glucose-starved cells expressing high SLC7A11 levels. Furthermore, reducing agents that attenuate disulfide stress (DTT, 2ME, and

TCEP) also appeared to inhibit this form of death.

Further mechanistic studies showed that intracellular disulfide stress could activate the Rac-WRC-Arp2/3 signaling pathway, causing the abnormal crosslinking of disulfide bonds between actin cytoskeleton proteins. This induced the collapse of the F-actin network, subsequently leading to cell death [10] (Fig. 1).

Studies have demonstrated that the morphological characteristics and biochemical processes of disulfidptosis are distinct from those of other forms of cell death. Importantly, evidence indicates that disulfidptosis may be involved in the development and prognosis of several diseases, including lung adenocarcinoma (LUAD) [14], bladder cancer (BCa) [15], renal cell carcinoma (RCC) [16], hepatocellular carcinoma (HCC) [17], and colon adenocarcinoma (COAD) [18]. Thus, disulfidptosis has emerged as a new hotspot and direction of research in the field of disease treatment. Hence, in this narrative review, we provide an updated summary of research on disulfidptosis, hoping to guide the exploration of its underlying mechanisms. Finally, based on these findings, we propose potential therapeutic approaches for conditions linked to disulfidptosis.

## 3. Connections between Disulfidptosis and Other Cell Death Pathways

The most significant morphological feature of cells undergoing disulfidptosis is the appearance of lamellipodial protrusions, likely resulting from abnormal actin cross-linking. This characteristic is sufficient to distinguish disulfidptosis from existing main modes of cell death, including ferroptosis (increased mitochondrial membrane density and decreased cristae, normal-sized nucleus), apoptosis (cell membrane bubbling, chromatin condensation, nuclear fragmentation), necroptosis (cell membrane perforation, organelle swelling, nuclear disintegration), autophagy (nuclear fragmentation, appearance of autophagosomes), pyroptosis (cell membrane perforation, chromatin condensation, appearance of pyroptosomes), and cuproptosis (mitochondrial shrinkage, cell membrane rupture, endoplasmic reticulum damage) (Table 1). Cells undergoing disulfidptosis require two key promoting factors: high expression of SLC7A11 and glucose starvation (GLUT inhibition). Current research indicates that these factors are indispensable, leading to the unique biochemical characteristics of disulfidptosis: depletion of NADPH and abnormal accumulation of disulfides such as cystine. Furthermore, the collapse of the F-actin network is a critical feature because it ultimately leads to cell death. This aspect also completely differs from other modes of cell death: ferroptosis (lipid peroxidation), apoptosis (engulfment and clearance by neighboring cells), necroptosis (cell membrane rupture), pyroptosis (cell swelling until rupture), and cuproptosis (protein toxic stress response) (Table 1). It’s not hard to see that both disulfidptosis and ferroptosis involve a critical factor: SLC7A11. In disulfidptosis, the accumulation of cystine relies on the transport activity of SLC7A11. In ferroptosis, the expression of SLC7A11 can promote the synthesis of glutathione, inhibiting lipid peroxidation and resisting the ferroptotic outcome in cells [19, 20]. Thus, the role of SLC7A11 seems to be opposite in disulfidptosis and ferroptosis. Additionally, both disulfidptosis and ferroptosis undergo intense oxidative stress processes, but the outcomes are markedly different: the former involves the accumulation of disulfides such as cystine and NADPH depletion, whereas the latter involves reactive oxygen species (ROS) accumulation and lipid peroxidation.

## 4. Molecular Mechanisms of Disulfidptosis

### 4.1. SLC7A11 in disulfidptosis

**Figure 2.**
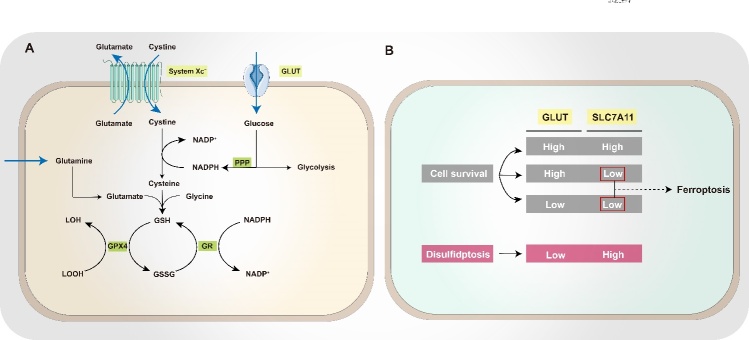
**Structure and function of SLC7A11**. (**A**) Extracellular cystine is taken into the cell through SLC7A11 and then transformed into cysteine by a reduction reaction that depletes NADPH. Following this, cysteine is converted into GSH in a two-step process. GPX4 employs GSH to reduce lipid hydroperoxides to lipid alcohols, with GSH being oxidized to GSSG. Subsequently, GSSG is reverted to GSH via a GR-mediated reduction, utilizing NADPH in the process. (**B**) Essential conditions for triggering disulfidptosis: glucose starvation/GLUT inhibition and overexpression of SLC7A11. Abbreviations: GPX4, glutathione peroxidase 4; GSH, reduced glutathione; GSSG, oxidized glutathione; LOOH, lipid hydroperoxide; LOH, lipid alcohol.

In studies on disulfidptosis, high SLC7A11 expression has been confirmed to be a prerequisite for cell death, as this protein mediates cystine uptake [10]. Excessive accumulation of cystine induces disulfidptosis in two ways: (1) consuming more NADPH until depletion (2) triggering disulfide stress. SLC7A11, also called xCT, belongs to solute carrier family 7. It is a transmembrane protein with 12 transmembrane domains, a cytoplasmic N-terminus, and a cytoplasmic C-terminus. Essentially, this protein is a cystine/glutamate antiporter, and it is highly expressed in most tumor cells [19, 35]. In conjunction with its partner protein SLC3A2, SLC7A11 typically forms system Xc^-^, which maintains the stability and appropriate membrane localization of SLC7A11 [36]. System Xc^-^ is primarily responsible for importing extracellular cystine and exporting intracellular glutamate at a 1:1 ratio [37]. Cancer cells often experience high levels of oxidative stress, necessitating cystine intake via SLC7A11 for the synthesis of glutathione (GSH), which can help to counteract oxidative damage [38]. SLC7A11 also has other functions, including the regulation of ferroptosis and the cell’s nutrient dependency [39-41] (Fig. 2). In summary, SLC7A11 serves as a critical regulatory factor, controlling metabolic reprogramming and the redox balance of the cell. Thus, it is required for cell survival and growth.

**Table 1:** Different modes of programmed cell death.

	Disulfidptosis	Ferroptosis	Apoptosis	Necroptosis	Autophagy	Pyroptosis	Cuproptosis
**Morphological Features**	Lamellipodial Protrusions	Cellular membrane bubbling Chromatin decondensation Normal nuclear size Increased mitochondrial membrane density and reduction in cristae	Cellular membrane blebbing Cellular contraction Chromatin condensation Nuclear rupture Organelle aggregation Appearance of apoptotic bodies	Cellular membrane perforation Cellular swelling Loss of chromatin Nuclear fragmentation Organelle swelling Appearance of necrotic bodies	Loss of cellular membrane specialization Possible presence of bubbling phenomena Cytoplasmic amorphousness Nuclear breakdown Organelle swelling Autophagic vacuoles Autolysosomes	Cellular membrane perforation Cellular flattening Chromatin condensation Nuclear condensation Damage to mitochondria and lysosomes Appearance of pyroptotic bodies	Mitochondrial shrinkage Cell membrane rupture Endoplasmic reticulum damage Chromatin disruption
**Biochemical Characteristics**	NADPH depletion Abnormal accumulation of cystine and other disulfides	Elevated ROS Accumulation of iron and reactive oxygen species GSH depletion Lipid peroxidation	DNA fragmentation Activation of apoptotic protease caspases Externalization of phosphatidylserine (PS) on the cell membrane	Declining ATP Levels RIP1, RIP3, and MLKL activation DAMP (e.g., HMGB1) release Overactivation of PARP1	Conversion of LC3-I to LC3-II and substrate (e.g., p62) degradation	Inflammasome Activation of caspases and gasdermin Release of a massive amount of pro-inflammatory cytokines	Increased levels of copper, pyruvate, alpha-ketoglutarate, and HSP70 Decreased levels of iron-sulfur (Fe-S) clusters
**Mode of Death**	Collapse of the actin cytoskeleton	Lipid peroxidation, cellular exhaustion	Engulfment and clearance by neighboring cells and phagocytic cells	Explosive rupture of the cell membrane	Autophagy, degradation, and digestion	Gradual swelling of the plasma membrane until rupture	Proteotoxic stress response
**Molecular Markers**	SLC7A11 Accumulation of cystine and other disulfides NADPH depletion	Elevated 4-HNE SLC7A11 GPX4 Ferritin	Activation of Caspase-3 Externalization of Phosphatidylserine (PS)	Elevated phosphorylation of RIPK1, RIPK3, and MLKL Reduced activity of Caspase-8	Atg1 (ULK1) Protein Kinase Complex Vps34 (PIK3C3)-Beclin1 (BECN1) complex PI3K Atg12-Atg5 complex LC3-PE conjugate	Activation of inflammasomes (such as NLRP3) Activation of Caspase-1 (cleaved state) Elevated GSDMD-N (activated state)	Lipoylated protein aggregation Reduced Fe-S cluster protein levels
**Signaling Pathways**	RAC1-WAVE-Arp2/3	System Xc^-^/GpX4 Pathway Lipid metabolism pathway Iron metabolism pathway	Extrinsic pathway (death receptor-mediated) Intrinsic pathway (mitochondria-mediated, endoplasmic reticulum pathway)	RIPK pathway Panx1 pathway	ULK1 pathway Beclin1 pathway AMPK pathway	Classical pyroptosis pathway (Caspase-1-dependent) Non-classical pyroptosis pathway (Caspase-4, 5, 11-dependent)	Activation of oxidative stress-related signaling pathways Disruption of the ubiquitin-proteasome system Targeting of lipoylated TCA cycle proteins
**Inflammatory Response**	N/A	Yes	No	Yes	No	Yes	N/A
**Caspase-Dependent**	Independent	Independent	Dependent	Independent	Independent	Dependent	Independent
**Effector Protein**	Actin	GPX4	Non-inflammatory caspases (Caspase-3, Caspase-6, and Caspase-7)	MLKL protein	LC3 Protein	Gasdermin Family	Lipoylated proteins
Inducers	Thiol oxidants (such as diamide and malathion) GLUT1 Inhibitor BAY-876 GLUT1/3 Inhibitor KL-11743	Erastin FIN56 Sorafenib RSL3 Cisplatin	TNF-α SM-164(TS) CCCP	TNF-α SM-164(TS) Z-VAD-FMK	Rapamycin EBSS	LPS+Nig DAMP/PAMP	Elesclomol (ES) Disulfiram (DSF) 2,2'-dithiodipyridine (DPy)
**Inhibitors**	DTT 2ME TCEP	Ferrostatin-1 SP600125 Liproxstatin-1	Z-VAD-FMK	Nel-1/3/5/7	Chloroquine 3-MA NH4Cl Bafilomycin A1	VX765 Disulfiram GSDME inhibitor	8-hydroxyquinoline (8-HQ) Tetrathiomolybdate (TTM)
**Inducing Factors**	Disulfide stress	Iron accumulation	Physiological and mild external stimuli	Pathological and strong external stimuli	Nutrient deficiency Hormone induction	Inflammasome activation	Copper accumulation
**Indicators**	Cell viability ATP levels ROS levels NADP+/NADPH ratio Glucose levels	ROS levels GSH and MDA contents Iron levels LPO GPX4	TUNEL Bcl-2 Annexin-V Mitochondrial membrane potential	Hexosaminidase Calcein-AM Annexin-V ATP levels	Levels of LC3 and ATG Series proteins	Caspase-1/4 Gasdermin D IL-18 IL-1β	Copper FDX1 DLAT LIAS HSP70.
**References**	[10]	[21-23]	[24, 25]	[26, 27]	[28-30]	[31, 32]	[33, 34]

Interestingly, research shows that cystine uptake via SLC7A11 can be both useful and detrimental to intracellular redox regulation. The elevated expression of SLC7A11 makes cells more reliant on glucose and the PPP [42, 43]. Moreover, in cancer cells, cystine uptake via SLC7A11 is crucial for maintaining the redox balance through GSH synthesis [44]. In fact, SLC7A11 is often upregulated in cancer cells while they adapt to oxidative stress [45-47]. Moreover, when SLC7A11 is inhibited or cystine uptake is blocked, GSH synthesis is reduced. These results in increased ROS levels and oxidative stress. Together, these effects can inhibit cancer cell growth [47]. The latest evidence shows that ferroptosis, characterized by iron accumulation and lipid peroxidation, also involves the inhibition of SLC7A11 activity. This leads to GSH depletion within cells, and lipid peroxides cannot be converted into hydroxyl compounds, inducing ferroptosis [22, 48-50]. Therefore, high SLC7A11 expression promotes the GSH synthesis by increasing cystine uptake. It thus counteracts oxidative stress and prevents ferroptosis, apoptosis, and necrosis, thereby protecting the cell. However, studies have found that high levels of SLC7A11 enhance the glucose dependence of cancer cells, exposing a potential vulnerability in cancer cell metabolism [51, 52].

It is well known that glucose is an essential energy source for cells. Glucose enters cells through glucose transporters (GLUTs) and is phosphorylated to glucose-6-phosphate by hexokinase (HK). This glucose-6-phosphate can enter one of two metabolic pathways: the glycolytic pathway or the PPP. Notably, the oxidative branch of the PPP produces NADPH, which helps in maintaining the redox balance by increasing cellular GSH stores. Furthermore, it also acts as a reducing agent for lipids, nucleotide, and amino acid biosynthesis. Cancer cells often undergo metabolic reprogramming, which makes them more reliant on metabolic substrates, such as glucose and more sensitive to glucose deprivation [53]. This phenomenon is especially evident in glioblastoma cells. Interestingly, SLC7A11 expression has been found to be upregulated in glioblastoma cell lines and the brains of glioblastoma patients [54, 55]. Notably, a western blotting experiment indicated that SLC7A11 expression is associated with the sensitivity of glioblastomas to glucose deprivation [56]. Further studies revealed that glioblastoma cells with high SLC7A11 expression are sensitive to GLUT1 depletion. In line with this finding, another study demonstrated that SLC7A11 overexpression results in extensive cell death in SK-BR-3 cells (human breast cancer cell line) following glucose withdrawal [57]. By contrast, studies have shown that in glioblastoma cells with high SLC7A11 activity, glucose metabolism can prevent the oxidative stress and cell death induced by SLC7A11-mediated cystine uptake [54, 55]. Likewise, in a glucose-rich medium, the proliferation rate of HeLa cells overexpressing SLC7A11 can remain unchanged [57]. Together, these findings suggest that the high SLC7A11 expression induces a “glucose addiction”-like state and heightens sensitivity to glucose starvation. Furthermore, both reductions in glucose supply and the inhibition of GLUT activity produce similar effects [10].

**Figure 3.**
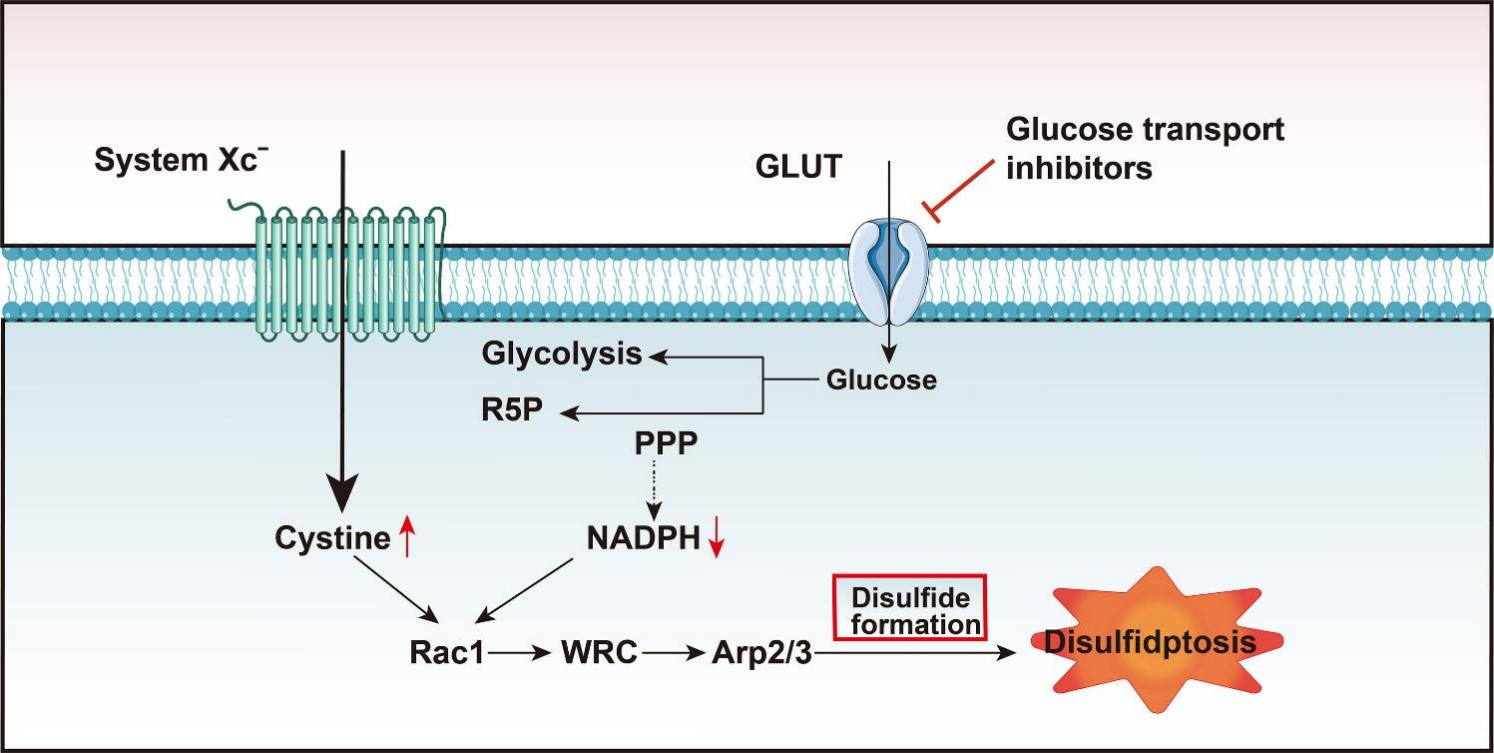
**Regulatory pathways of disulfidptosis**. In SLC7A11-overexpressing cells, treatment with GLUT inhibitors causes intracellular cystine accumulation and NADPH consumption. Additionally, disulfide bonds between actin molecules are generated via the Rac/WRC/Arp2/3 pathway. This eventually results in cell death. Abbreviations: PPP, pentose phosphate pathway; R5P, ribulose-5-phosphate; NADPH, nicotinamide adenine dinucleotide phosphate; WRC, WAVE regulatory complex; Arp, actin-related protein.

The next mystery lies in how SLC7A11 overexpression specifically enhances glucose dependence. Cells undergoing glucose deprivation-induced death display the intracellular accumulation of L-cysteine and its oxidized dimer, L-cystine. Interestingly, T98 cells (human glioblastoma cell line) starved of glucose for 8 hours in cystine-free medium can maintain their viability. However, the addition of L-cystine can induce cell death under glucose deprivation [56]. Mechanistically, this is reasonable, as one primary function of SLC7A11 includes cystine uptake. Therefore, SLC7A11 overexpression results in increased cystine uptake; subsequently, the accumulation of cystine can induce cytotoxicity, thus promoting cell death. However, the inhibition of SLC7A11 activity through drugs such as sulphasalazine (SASP) or erastin can prevent glucose deprivation-induced cell death. One study showed that treatment with SASP can prevent L-cysteine and L-cystine accumulation following glucose deprivation [56]. Moreover, the inhibition of SLC7A11 using CRISPRi. (40) (dCas9-KRAB) was also found to rescue cells from glucose starvation [10]. This evidence corroborates that cystine accumulation via SLC7A11-mediated transport is a significant contributor to cell death. In addition, it also confirms that SLC7A11 overexpression is a crucial prerequisite for cell death in glucose-starved cells. Studies demonstrating improved cell vitality during glucose withdrawal in cells depleted of Nrf2 (which results in reduced SLC7A11 expression) further substantiate this point [57]. Moreover, it has been shown that intracellular NADPH levels plummet rapidly under glucose starvation, with this timeframe being akin to that required for cysteine and cystine accumulation post-glucose deprivation [56]. This is not surprising, given that intracellular cystine reduction is NADPH-dependent. This puts NADPH in a “double-bind” under conditions of glucose deprivation, as its production via the PPP reduces due to glucose unavailability and it is also extensively consumed for reducing cystine. Eventually, NADPH depletion occurs and cystine continues to accumulate, resulting in cytotoxicity. Notably, other studies have shown that both glucose addition and reduced SLC7A11 expression can completely inhibit cystine-induced NADPH consumption [57, 58]. Overall, these phenomena suggest that under conditions of glucose starvation, cells with high SLC7A11 expression are heavily reliant on glucose (primarily through the PPP). However, excessive cystine accelerates NADPH consumption, leading to a subsequent collapse of the redox balance and cell death (Fig. 3).

### 4.2. Cystine in disulfidptosis

In the previous section, we discussed how high SLC7A11 expression is a prerequisite for disulfidptosis. In essence, it facilitates the increased influx of cystine, and the accumulation of intracellular cystine is a critical factor in disulfidptosis.

**Figure 4.**
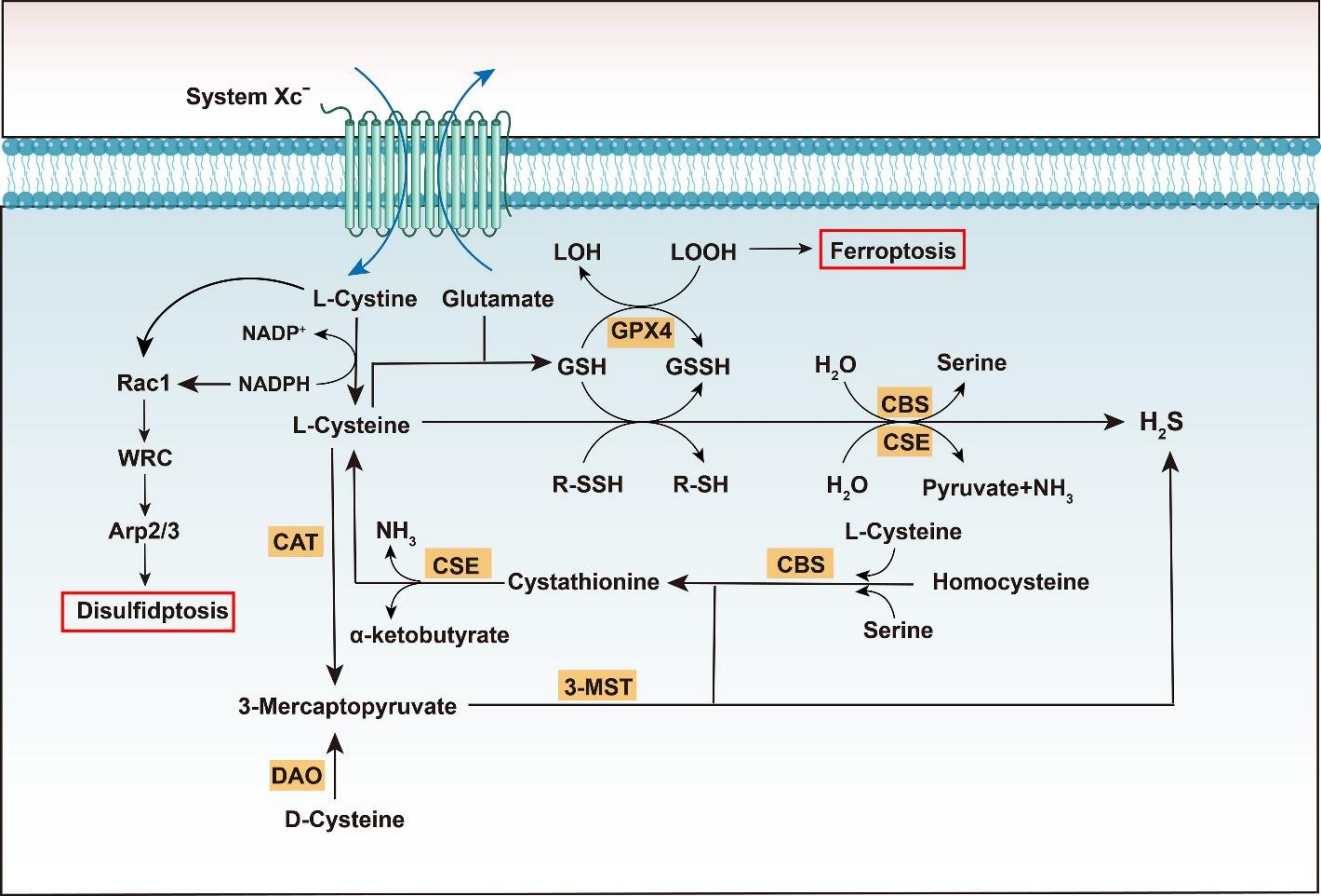
**Relationship of sulfide metabolites with disulfidptosis and ferroptosis**. Cysteine is a crucial intermediate product in intracellular sulfur metabolism. On one hand, cysteine is generated to produce hydrogen sulfide (H_2_S) through the catalytic actions of cystathionine-γ-lyase (CSE) and cystathionine-β-synthase (CBS), or via the pathway involving cysteine aminotransferase (CAT) and 3-mercaptopyruvate sulfurtransferase (3-MST). On the other hand, cysteine metabolism is intricately linked to processes such as disulfidptosis and ferroptosis: the accumulation of cystine contributes to disulfidptosis; while glutathione, synthesized from cysteine, can, under the influence of GPX4, reduce lipid hydroperoxides to lipid alcohols, thereby inhibiting ferroptosis. Abbreviations: GPX4, glutathione peroxidase 4; GSH, glutathione; GSSG, oxidized glutathione; LOH, Lipid Alcohols; LOOH, Lipid Hydroperoxides; NADPH, nicotinamide adenine dinucleotide phosphate; WRC, WAVE regulatory complex; Arp, actin-related protein; 3-MST, 3-mercaptopyruvate sulfurtransferase; CAT, cysteine aminotransferase; CBS, cystathionine-β-synthase; CSE, cystathionine g-lyase; DAO, D-amino acid oxidase.

In the human body, cystine (two cysteine molecules joined by a disulfide bond form one cystine molecule) is the principal form of sulfur. It is key for the synthesis and metabolism of various substances such as proteins and sulfur-containing molecules (e.g., GSH and Coenzyme A) [59, 60] (Fig. 4). Due to its propensity for oxidation, cysteine predominantly exists as cystine extracellularly [61]. However, cystine is transported intracellularly by system Xc^-^ in a Na^+^-independent manner [62, 63]. In specific cells, such as neurons, cystine is directly taken up by excitatory amino acid transporter 3 (EAAT3/EAAC1). Due to the reductive intracellular environment, the imported cystine is swiftly reduced to cysteine via GSH or thioredoxin reductase 1 (TRR1). Notably, large amounts of NADPH are consumed during this process [64]. Cysteine can be further converted to GSH via reactions with glutamate and glycine, which are catalyzed by glutamate-cysteine ligase (GCL) and glutathione synthetase (GS) in the cytoplasm. Thus, cysteine is considered the limiting substrate for GSH synthesis [65-67]. The average concentration of GSH in the cytoplasm is 1-11 mM. Moreover, GSH performs various functions, especially in the redox buffering system, where it plays the most crucial role of regulating the GSH-GSH disulfide (GSH/GSSG) redox couple [61, 68] (Fig. 4). GSH can reduce disulfide bonds and hydrogen peroxide (H_2_O_2_) through glutathione peroxidase (GPx), mitigating oxidative stress. Concurrently, glutathione reductase (GR) utilizes the electrons from NADPH to reduce disulfides into dithiols, further reducing oxidized GSH (disulfide GSSG) into reduced GSH [69]. Accordingly, a stable GSH/GSSG ratio is vital for the maintenance of cellular redox homeostasis and biosynthesis. Hence, a series of metabolic products derived from cystine are crucial for maintaining cellular homeostasis.

During disulfidptosis, there is an imbalance of cystine levels. This induces disulfide stress and culminates in cell death [10]. The regulatory role of cystine in cells and tumors has long been explored. Current research indicates that both the deficiency and accumulation of cystine can impair cell survival to a certain extent. Cystine starvation can reduce the expression of glutathione peroxidase 4 (GPX4) by inhibiting mTORC1/4E-BP1-mediated protein translation. Moreover, it can decrease intracellular GSH levels, synergistically inducing ferroptosis [70, 71]. Additionally, cystine deprivation can trigger rapid necrosis in von Hippel-Lindau (*VHL*)-deficient RCC [72].

Concerning the impact of cystine accumulation, cystinuria has received extensive attention in previous studies. Cystinuria is an autosomal recessive metabolic disorder caused by mutations in the gene encoding cystinosin (CTNS). Moreover, it is characterized by the abnormal accumulation of cystine (the oxidized form of cysteine) in lysosomes [73-75]. The accumulation of cystine crystals in lysosomes is also a marker of a kidney disease called cystinosis [76, 77]. Notably, studies have revealed that the intake of L-cystine significantly promotes ROS accumulation, affecting the cellular redox balance [78, 79]. These studies exploring cystine levels provide important insights for cancer treatment. Likewise, studies on disulfidptosis show that this novel form of cell death caused by cystine accumulation could potentially become an important target for cancer treatment, allowing us to circumvent the unique evasion mechanisms of drug-resistant tumor cells.

### 4.3. NADPH in disulfidptosis

The depletion of NADPH is a hallmark metabolic event in disulfidptosis and conveys a critical signal. That is, it shows that the cells undergoing disulfidptosis are experiencing some degree of redox imbalance (for example, the abnormal accumulation of disulfides). Specifically, as NADPH is depleted, the accumulation of cystine and other disulfides cannot be cleared. This ultimately leads to an imbalance in the redox environment and the collapse of the cytoskeleton.

The physiological role of NADPH is primarily attributed to its strong reducing power [80, 81]. Using NADPH, disulfides can be converted to thiols under the action of GR and thioredoxin (Trx) reductase (TrxR). During these reactions, oxidized GSH (disulfide GSSG) is converted into reduced GSH, and Trx-(SH)_2_ is reduced to Trx. GSH can reduce disulfide bonds and peroxides through GPx to mitigate ROS toxicity. Moreover, it can promote the glutathionylation of cysteine residues via glutathione S-transferase (GST) to protect proteins from oxidation [82]. Trx also promotes the peroxiredoxin (Prx)-mediated decomposition of H_2_O_2_ into H_2_O and O_2_, Additionally, it maintains the activity of ribonucleotide reductase through its reductive capabilities, thereby enabling DNA synthesis [83, 84].

Lipid metabolism and oxidative stress have a crucial impact on the survival of cancer cells. NADPH, as a coenzyme, is responsible for maintaining the lipid and redox balance in cancer cells, while also participating in reductive biosynthesis [85]. For instance, in pancreatic cancer cells, NADPH and NADP+ stores increase significantly during oxidative stress [86]. In fact, cancer cells have evolved a set of systems to counteract the detrimental impact of oxidative stress. For example, some studies have found that glucose-6-phosphate dehydrogenase (G6PD) deficiency increases NADP^+^ levels. This causes a compensatory increase in the ME1 and IDH1 flux, alleviating the pressure created by decreased NADPH levels [87, 88]. Additionally, under NADPH-deficient conditions, AMPK can inhibit fatty acid synthesis by suppressing ACC1 and ACC2 activity, thereby reducing NADPH consumption [89]. Moreover, TAp73 is overexpressed in cancer cells, increasing the PPP flux to generate more NADPH for ROS detoxification and macromolecule synthesis [90]. However, if these remedial mechanisms fail to eliminate or attenuate NADPH deficiency, ROS levels may increase to a critical threshold. This may induce cytotoxicity and cause genetic damage and death. These findings underscore the strong dependency of cancer cells on the reducing power of NADPH.

NADPH is derived from NAD+. NAD+ is converted to NADP+ via NAD kinase (NADK) or NADK2. Subsequently, NADP+ is further reduced to NADPH via the PPP, malic enzyme (ME)-mediated malate metabolism, folate metabolism, and isocitrate dehydrogenase (IDH)-mediated isocitrate/α-ketoglutarate (α-KG) metabolism [85, 88]. The PPP is crucial for NADPH generation in cancer cells. This is because, in proliferating cells, the most important source of cytosolic NADPH is the PPP. Accumulating evidence shows that many human cancers have a higher PPP flux, which allows them to produce more NADPH. G6PD, the key enzyme of this pathway, is overexpressed in various types of cancers, including bladder, breast, prostate, and stomach cancer, among other types of malignancies. Its elevated expression is also associated with a poor prognosis in patients with these cancers [80, 91, 92]. Other key enzymes such as transketolase 1 (TKTL1) and transaldolase (TALDO) are also often overexpressed in cancers [93]. Concurrently, in cancer cells with high NADPH consumption, the activity of G6PD is significantly enhanced to compensate for NADPH depletion [94]. Conversely, G6PD depletion significantly reduces NADPH levels, disrupting the redox balance. Similarly, the knockdown of 6-phosphogluconate dehydrogenase (6PGD), another enzyme required for the PPP, also inhibits NADPH production. This inhibition is sufficient to suppress cell growth and lactate production in some types of cancers and can also prevent metastasis [86, 92, 95].

Notably, these phenomena are orchestrated by specific cellular mechanisms. G6PD, as a rate-limiting enzyme, significantly influences the flux distribution between glycolysis and the PPP. G6PD activity also determines the yield of NADPH to a certain extent. Therefore, to meet their metabolic and bioenergetic requirements, proliferating cancer cells activate G6PD to increase the PPP flux and obtain more NADPH and other resources. Additionally, the oxidative branch of the PPP appears to be essential for maintaining a normal NADPH/NADP ratio, mammalian dihydrofolate reductase (DHFR) activity, and folate metabolism [88]. When NADP+ is elevated, it competes with NADPH for binding to G6PD and thus activates this enzyme. This is meaningful for cancer cells, as they experience highly oxidative environments, which can increase their NADP+/NADPH ratio. The increased NADP+ levels in turn activate G6PD and the PPP, generating NADPH to compensate for redox stress. In conclusion, the NADPH produced through the PPP is crucial for the survival and development of cancer cells.

### 4.4. Disulfide bonds in disulfidptosis

A deficiency of NADPH leads to the transient elevation of cystine and the accumulation of disulfides. These factors are direct inducers of cell death [10] (Fig. 3). This is also a key phenomenon in disulfidptosis.

Indeed, disulfide bonds have multiple critical physiological roles in organisms. For example, they aid in stabilizing protein structures, modulating protein functions, transmitting cell signals, ensuring cell adhesion, and assembling matrixes. Moreover, disulfide compounds are crucial regulators of the redox balance, which is vital for maintaining normal physiological functions [59, 60]. Disulfide bonds are generally formed via the oxidation of two adjacent thiol groups, and they are typically inert and stable. The cytoplasm is a highly reductive environment. Therefore, the formation of disulfide bonds does not usually occur in the cytoplasm. In fact, the disulfide bonds required for protein synthesis are commonly generated in the endoplasmic reticulum [96-98]. However, under conditions of stress, cysteine may form disulfide bonds in the cytoplasm via several pathways. These include cysteine oxidation, glutathionylation, and reactions between cysteine thiols exposed to ROS-formed sulfenic acid (RSOH) and GSH or reactions between neighboring cysteines [99]. These cysteine disulfides are critical for controlling the cellular redox state, which is maintained through cycles of disulfide bond formation and cleavage. For instance, GSSG/GSH often controls the generation and reduction of cellular disulfides via thiol-disulfide exchange reactions. Additionally, the metabolism related to disulfide bonds is equally important in the growth and development of cancer cells. For example, prolyl 4-hydroxylase subunit beta (P4HB) is upregulated in most tumors, catalyzing the formation, breakage, and rearrangement of disulfide bonds through two thioredoxin domains [100]. Hence, it is clear that protein thiol disulfides play a crucial regulatory role in biological systems.

However, the excessive accumulation of disulfide compounds can trigger disulfide stress and induce disulfidptosis. Previous research has shown that the import of L-cystine (not cysteine) promotes ROS accumulation, GSH depletion, and redox imbalance in cancer cells under conditions of glucose starvation. Notably, this is accompanied by NADPH depletion [56, 57, 101]. Additionally, the generation of mixed disulfides from cysteine and GSH leads to actin oxidation, and actin glutathionylation is critical for cell spreading and cytoskeletal organization [102]. Some studies have also demonstrated that H_2_O_2_ can increase the amount of polymerized actin (F-actin) within cells. Subsequently, disulfide-bonded actin dimers are incorporated into F-actin during polymerization, producing cross-linked actin filaments [103, 104]. These findings corroborate the conclusions reached by Liu et al. Specifically, they demonstrate that disulfide stress in glucose-starved cancer cells expressing high SLC7A11 levels can cause the abnormal cross-linking of cytoskeletal proteins via disulfide bonds, inducing cell death through the collapse of the F-actin network [10]. Notably, ROS scavengers cannot prevent this form of cell death. Moreover, experiments in GSH-depleted cells have demonstrated that neither the loss of GSH nor the absolute levels of GSSG generated during exposure to oxidants are the cause of actin polymerization under these conditions [105]. This suggests that disulfidptosis is not directly caused by ROS accumulation or GSH depletion. In fact, these changes only reflect a series of metabolic events orchestrated by the redox imbalance during disulfidptosis. Overall, based on current evidence, it is clear that the accumulation of disulfide bonds directly prompts this novel pathway of cell death. Moreover, treatment with reducing agents that prevent disulfide stress can inhibit disulfidptosis.

### 4.5. Rac-WRC-Arp2/3 in disulfidptosis

Studies on disulfidptosis have often reported that cells undergoing programmed death exhibit lamellipodia formation. Notably, the Rac-WRC-Arp2/3 signaling pathway has been implicated in this process (Fig. 3). Therefore, understanding this pathway is crucial for achieving a comprehensive understanding of disulfidptosis.

**Figure 5.**
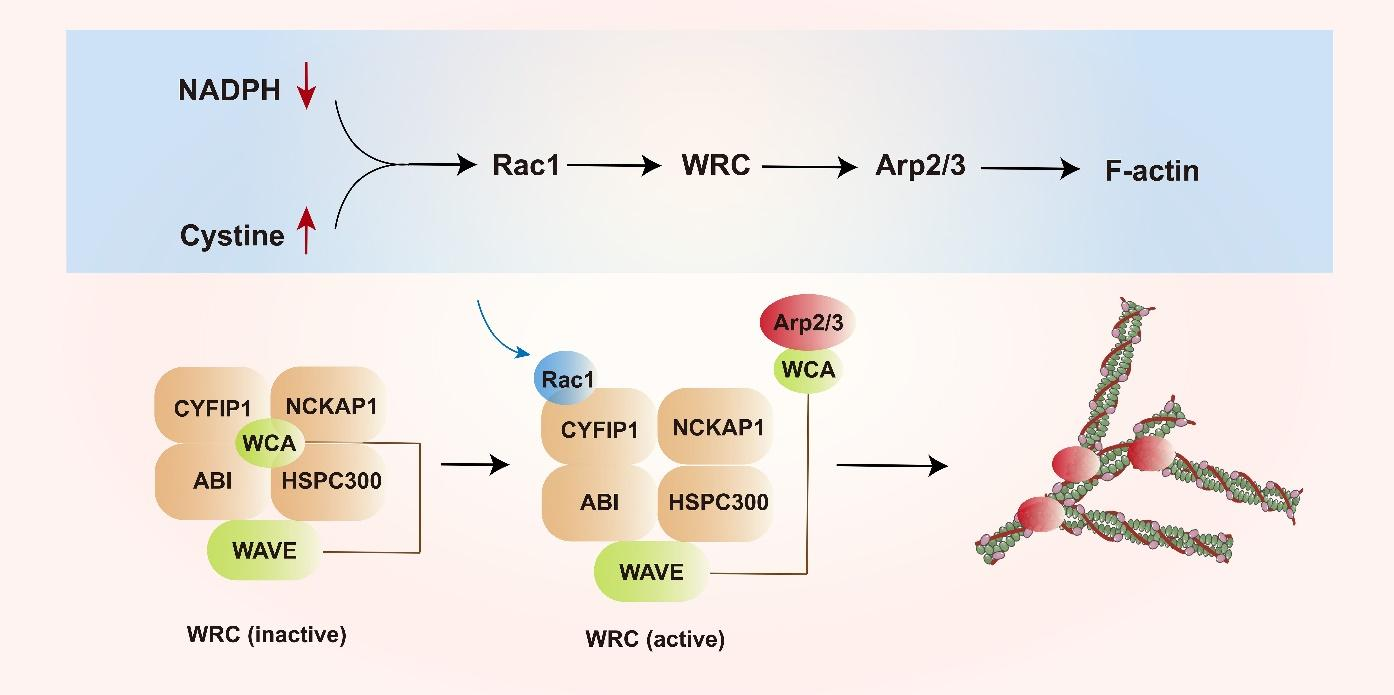
**Composition of the WRC and the activity of the Rac1-WRC-Arp2/3 signaling pathway**. WRC is a five-subunit complex, including the protein families: ABI (ABI1, ABI2, or ABI3), WAVE (WAVE1, WAVE2, or WAVE3), NCKAP1, CYFIP (CYFIP1 or CYFIP2), and HSPC300. In the absence of Rac1 binding, the WRC is in an inhibited state. Binding of Rac1 to CYFIP1 induces WRC activation, triggering the release of the WCA domain, thereby activating Arp2/3 and contracting F-actin. Abbreviations: Rac1, Ras-related C3 Botulinum Toxin Substrate 1; Arp2/3, Actin-related Protein 2/3; ABI, Abl-interactor; NCKAP1, NCK-associated protein 1; CYFIP1, Cytoplasmic FMR1-interacting protein 1; HSPC300, Hematopoietic stem/progenitor cell protein 300.

Several studies have proven that the Rac-WRC-Arp2/3 pathway is involved in regulating cancer cell migration. The migratory ability of cancer cells is critical for cancer metastasis. Actin-related protein 2/3 complex (Arp2/3) — a stable assembly of seven protein subunits — plays a pivotal role in cell migration [106-110]. Cancer cell migration involves several steps: the reduction of cell adhesion, rearrangement of the cytoskeleton, degradation of the extracellular matrix, and development of protrusions and pseudopodia. Specifically, pseudopodia can be categorized into filopodia, lamellipodia, invasive pseudopodia, and podosomes. In cancer cells, lamellipodia are the most characteristic type of cellular protrusions [111, 112]. It is widely believed that lamellipodia are actin-filled membrane sheets that generate forces. These forces allow cell motility via matrix attachment, thus promoting cell migration [113]. Hence, lamellipodia appears to arise due to localized actin aggregation. The Arp2/3 complex orchestrates this process by nucleating actin cores through ATP hydrolysis [114]. Thus, the Rac-WRC-Arp2/3 pathway induces cell death by promoting actin contraction and is currently the only pathway implicated in disulfidptosis [10, 115, 116] (Fig. 5). Furthermore, research has summarized the interactions between mitochondrial dynamics and the cytoskeleton, attempting to connect mitochondria, the cytoskeletal system, and membranous organelles into a cohesive whole [117]. Since mitochondria frequently play a crucial role in metabolism related to cell death, including in the recent focus on ferroptosis [118], paying attention to mitochondrial activity while concentrating on the cytoskeleton's role in disulfidptosis could provide new insights.

Given that disulfidptosis was only identified as a form of cell death in recent times, research in this field is currently in its nascent stage. Hence, it is unclear how Rac-WRC-Arp2/3 ultimately induces cell death and whether other upstream and downstream molecules participate in this pathway. Hence, more research is warranted to delineate this process. Besides the above-mentioned metabolic mechanisms of disulfidptosis, focusing on the roles of related genes is also crucial. Indeed, some genetic underpinnings of disulfidptosis have been discovered in recent studies, and these genes will be featured prominently in the following section.

## 5. Genes Involved in Disulfidptosis

This section discusses the relevant genes involved in the process of disulfidptosis, focusing on their respective roles (Table 2). Since the important functions of SLC7A11 and the related metabolism involved have been emphasized earlier, we will not elaborate further. We will mainly focus on introducing several significant related genes.

### 5.1. NCKAP1

*NCKAP1* encodes an important protein that binds to a protein complex known as the WASF regulatory complex, which has been shown to induce cell invasion and regulate the actin cytoskeleton [144]. Moreover, *NCKAP1* also participates in the formation of lamellipodia. Research has shown that *NCKAP1* is highly expressed in tumor cells and its expression levels are linked to the prognosis of diseases such as breast, prostate, and colon cancer [145]. Knocking out the *NCKAP1* gene markedly suppresses the migratory ability of cancer cells, likely due to a disruption of the WASF3 complex (WASF3 requires *NCKAP1* to facilitate invasion and metastasis). Reports indicate that the binding of the NCKAP1 protein to the cellular chaperone heat shock protein 90 (HSP90) is essential for its protein stability, suggesting that HSP90 is also involved in cell invasion and migration [146]. However, the high expression of *NCKAP1* in cancer cells does not solely contribute to negative outcomes; instead, it appears to exert a positive effect on treatment outcomes in some cancers. Studies have shown that *NCKAP1* regulates the cell cycle by targeting Rb1/p53, which is associated with a good prognosis in patients with HCC [147]. In human kidney cancer cells (ACHN), the overexpression of *NCKAP1* decreases the cells' invasive and migratory capabilities of the tumor cells, thereby suppressing cancer progression [148]. These results are promising, as the expression of *NCKAP1* may promote disulfidptosis. If the above mechanisms can synergize with disulfidptosis, this could offer a potential target for the treatment of related cancers.

### 5.2. PDLIM1

*PDLIM1* is a member of the PDZ-LIM family, and it is involved in various physiological processes, including cytoskeletal regulation and synapse formation. A large body of evidence indicates that *PDLIM1* is dysregulated in various types of cancers, contributing to tumor development and growth [127]. In colorectal cancer, the absence of *PDLIM1* promotes the expression of epithelial-mesenchymal transition (EMT) markers and enhances the invasive and migratory characteristics of tumor cells. Conversely, enhancing the expression of *PDLIM1* can inhibit the metastasis of CRC. This is because PDLIM1 exerts its effects by inhibiting the transcriptional activity of β-catenin, thereby preventing EMT in CRC [126]. Moreover, *PDLIM1* expression is notably downregulated in metastatic HCC tissues, suggesting its role in inhibiting metastasis. Additionally, the loss of *PDLIM1* in HCC cells results in the excessive production of F-actin, which is associated with disulfidptosis [149,150]. Nevertheless, in other types of cancers (e.g., breast cancer and chronic myelogenous leukemia), *PDLIM1* can promote cancer progression [151]. Overall, the evidence indicates that *PDLIM1* possesses distinct tissue-specific effects in different cancer types, likely by regulating different signaling pathways and targets. Intriguingly, the overexpression of *PDLIM1* has also been shown to attenuate the cancer-promoting function of miR-187 in gastric cancer (GC) cells and increase their sensitivity to cisplatin [152,153]. From this perspective, *PDLIM1* is a promising target for the treatment of various cancers, including its deep relationship with the molecular mechanisms and disulfidptosis.

**Table 2:** Genes involved in disulfidptosis.

Gene	Full name	Function	Related disorders	Subcellular locations	Ref.
***SLC7A11***	Solute carrier family 7 member 11	It mediates the exchange of extracellular anionic L-cystine and intracellular L-glutamate across the plasma membrane.	Drug addiction Schizophrenia Neurodegenerative disorders	Plasma membrane	[19, 51]
***RPN1***	Ribophorin I	It serves as a component of the N-oligosaccharyl transferase complex that links high mannose oligosaccharides to the asparagine residues found in the Asn-X-Ser/Thr consensus motif of nascent polypeptide chains.	Psoriasis-like disease Prostate cancer	Endoplasmic reticulum Cytosol	[119]
***NCKAP1***	Nck-associated protein 1	It is a constituent of the WAVE complex, and it is instrumental in the regulation of lamellipodia formation.	Pancreatic cancer Lung cancer	Cytosol	[120, 121]
***INF2***	Inverted formin 2	It mediates mitochondrial division and contributes to the assembly of stress fibers. It facilitates the initiation and extension of actin filaments and promotes filament disassociation via cleavage.	Focal segmental glomerulosclerosis (FSGS) Charcot-Marie-Tooth (CMT) disease	Endoplasmic reticulum Nuclear bodies	[122, 123]
***CD2AP***	CD2-Associated Protein	It exerts a pivotal regulatory effect on the actin cytoskeleton architecture. It is involved in the dynamic reorganization of actin filaments and membrane transport processes associated with receptor internalization and cell division.	Glomerular disease	Membrane ruffles Lipid rafts leading edges of cells	[124, 125]
***PDLIM1***	PDZ And LIM Domain 1	It is involved in regulating cytoskeletal dynamics and synapse formation.	CRC HCC	Actin stress fibers	[126, 127]
***ACTN4***	Actinin Alpha 4	It modulates the stability of the cytoskeletal network and sustains the integrity of the renal glomerular filtration barrier.	FSGS	Nucleus and cytoskeleton (actin filaments)	[128, 129]
***MYH9***	Myosin Heavy Chain 9	It participates in cellular adhesion processes, orchestrates cell migration, and contributes to the establishment of tissue structure. It is also essential for a spectrum of mechanical cellular functions, including cytoskeletal contractility.	May-Hegglin anomaly Fechtner syndrome Sebastian syndrome Epstein syndrome DFNA17.	N/A	[130-132]
***MYH10***	Myosin Heavy Chain 10	It engages in intercellular and cell-extracellular matrix adhesion, directs cellular migration and polarsity, and induces apoptosis in embryonic stem cells.	Left atrial enlargement	Adherens junctions within intercalated discs	[133]
***IQGAP1***	IQ Motif Containing GTPase Activating Protein 1	It is involved in the structuring of the actin cytoskeleton. It also modulates transcription, facilitates cellular adhesion, and regulates tshe cell cycle.	Aggressive colorectal ovarian cancers	Nucleus Plasma membrane Cytoplasm	[134]
***FLNA***	Filamin A	It regulates the reorganization of the actin cytoskeleton.	Filaminopathy A	Plasma membrane Actin filaments Cytosol	[135]
***FLNB***	Filamin B	It regulates intracellular communication and signaling by cross-linking the protein actin.	Skeletal deformities	Plasma membrane Golgi apparatus Actin filaments Cytosol	[136]
*TLN1*	Talin 1	It mediates cell-cell adhesion via the linkage of integrins to actin filaments.	Capillary leak syndrome Leukocyte adhesion deficiency	Focal adhesion sites Cytosol	[137, 138]
***MYL6***	Myosin Light Chain 6	It is involved in calcium ion binding and the structural constitution of muscle.	Alzheimer's disease	N/A	[139]
***ACTB***	Actin Beta	It is involved in cell motility, structure, integrity, and intercellular signaling.	lung, breast, prostate, ovarian cancers	N/A	[140]
***DSTN***	Destrin (Actin Depolymerizing Factor)	It breaks down actin filaments (F-actin) and binds to actin monomers (G-actin).	Rectal Cancer Lung cancer	Plasma membrane	[141, 142]
***CAPZB***	Capping Actin Protein of Muscle Z-line Beta Subunit	It is involved in the formation of the barbed ends of the rapidly growing actin filaments in the dynactin complex and stabilizes the structure of dynactin.	epithelioid sarcoma	Nucleoplasm Cytosol and vesicles	[143]

### 5.3. MYH9

The *MYH9* gene is responsible for producing the heavy chain of non-muscle myosin IIA (NMIIA). Thus, it plays a vital role in cell adhesion and is essential for the coordination of cell migration and the contractility of the cytoskeleton [130]. Current evidence shows that the effect of *MYH9* on cancer is dualistic, and this gene can have different effects even in the same type of cancer. Researchers have discovered that *MYH9* plays a tumor-suppressing role in mouse skin squamous cell carcinoma and melanoma [154]. However, in mouse head and neck squamous cell carcinoma, *MYH9* can have both a cancer-promoting and cancer-inhibiting role, and no definitive consensus regarding its mechanism of action exists [155,156]. Meanwhile, *MYH9* promotes the growth of other cancers, such as HCC, pancreatic cancer, gastric cancer, colorectal cancer, and breast cancer, and is associated with a poor prognosis in these patients. Furthermore, aminated fullerene derivative (C70-EDA) can bind to the C-terminal of *MYH9*, regulating its subcellular distribution and blocking *MYH9*-related cell motility [157]. These findings could help in the development of future cancer treatments based on disulfidptosis. Our hypothesis is that cells experiencing disulfidptosis with increased expression of *MYH9* could result in detrimental cancer outcomes, something we wish to avoid. Therefore, it is necessary to find a way to inhibit its tumor-promoting effects without affecting its expression.

### 5.4. FLNA

Filamin A (*FLNA*) is a widely recognized actin crosslinking protein, as well as the most numerous and broadly distributed member of the filamin family (FLNB and FLNC). *FLNA* plays a critical role in regulating the reorganization of the actin cytoskeleton. Recently, the debate over whether *FLNA* is a tumor-suppressing or cancer-promoting gene has garnered widespread attention. Intriguingly, the role of *FLNA* in cancer development depends significantly on its cellular localization. Specifically, FLNA promotes cancer progression through its interactions with signaling molecules, often leading to adverse outcomes. Conversely, when *FLNA* is situated in the nucleus, it inhibits cancer cell development and migration by either binding with transcription factors or diminishing activity in DNA promoter regions [158,159]. The overexpression of *FLNA* also follows the above pattern. Therefore, *FLNA* is likely to become a target for the treatment of disulfidptosis-related diseases, by attempting to develop drugs that target *FLNA* and localize it within the nucleus.

### 5.5. ACTB

*ACTB* is a highly conserved cytoskeletal structural protein involved in numerous aspects of cell growth, division, movement, and cytoskeletal formation. It has been reported that *ACTB* is highly expressed in most tumor cells, which is inseparable from the cytoskeletal changes and cell migration it induces, such as in renal cell carcinoma, gastric cancer, breast cancer, etc. However, in esophageal cancer, *ACTB* has been found to be downregulated at the RNA level, suggesting that *ACTB* may act as a suppressor in esophageal cancer [140]. Importantly, in three adenocarcinoma cell lines with high metastatic potential and invasiveness, both *ACTB* and F-actin expression levels were increased, promoting the formation of lamellipodia, consistent with their high metastatic ability [160]. Considering the special relationship between *ACTB* and F-actin in certain cancer cells, *ACTB* could emerge as a crucial target in disulfidptosis.

### 5.6. Other genes

In addition to the previously mentioned genes, the remaining genes identified in the table (such as *RPN1*, *INF2*, *CD2AP*, *ACTN4*, *MYH10*, *IQGAP1*, *FLNB*, *TLN1*, *MYL6*, *DSTN*, *CAPZB*) are observed to be upregulated in a range of tumor cells and play roles in regulating cytoskeletal activity and cell migration. Unfortunately, in current research, these genes primarily appear as promoters of cancer development and metastasis, which contradicts our hopes for inducing cell death through disulfidptosis. However, this is far from a pessimistic view; for instance, the expression of these genes could be an emergency response by cancer cells to counteract disulfidptosis (some form of metabolic reprogramming). In fact, much of the research on these genes is still in the preliminary stages. Consequently, further exploration is needed regarding the mechanisms, signaling pathways, and their connections with cancer development and progression concerning these genes. This will offer substantial perspectives and aid in delving into the mechanisms of disulfidptosis and its utilization in cancer therapy.

## 6. Disulfidptosis and Cancer - Treatment Implications

Programmed cell death is significant in the context of cancer occurrence, development, and treatment. Tumor growth and proliferation can be effectively inhibited by inducing programmed cell death and activating tumor-related immune responses to control cancer progression. In previous studies, elevated SLC7A11 levels in cancer cells have been linked to tumor formation, drug resistance, and poor prognoses. Specifically, this association has been observed in breast cancer, lung cancer, pancreatic cancer, and RCC [101]. SLC7A11 overexpression is believed to promote cancer growth and metastasis by modulating the tumor microenvironment and increasing cell proliferation. Meanwhile, cancer cells also exhibit intrinsic anti-apoptotic properties. Thus, several attempts have been made to induce cancer cell apoptosis to combat cancer. However, the effectiveness of these strategies has been very limited [161, 162]. Disulfidptosis, as a newly discovered programmed cell death pathway, could provide new opportunities for targeted cancer therapies.

### 6.6. Potential drugs (Traditional therapy)

#### 6.6.1. Targeting SLC7A11

As previously discussed, high SLC7A11 expression and the cystine transport mediated by this protein is crucial for disulfidptosis. Therefore, drugs that can promote SLC7A11 expression or function could be important from a cancer therapy perspective. In mouse peritoneal macrophages, the activity of system Xc- can be increased through various agents, including diethyl maleate (DEM), sodium arsenite, cadmium chloride, H_2_O_2_, bacterial lipopolysaccharide, and TNF-1 [163]. Notably, one study showed that Muller cells treated with DEM (an electrophilic reagent, which reduces intracellular cysteine and GSH levels), exhibit diminished levels of both GSH and cysteine. These conditions subsequently induce SLC7A11 expression and promote L-cystine uptake in these cells. This demonstrates that the reduction of cysteine and GSH is an important regulator of SLC7A11 activity, although the level of cysteine appears to be more critical in this aspect.) Additionally, the oxidative stress induced after DEM treatment is another non-negligible promoting factor.

Unlike DEM, H_2_O_2_ increases the nuclear translocation of Nuclear factor erythroid 2-related factor 2 (NRF2, detailed below), thereby upregulating the mRNA and protein levels of SLC7A11 [164,165]. Importantly, recent studies have discovered that high levels of SLC7A11 markedly promote H2O2-induced cell death. In other words, high SLC7A11 expression combined with H_2_O_2_ treatment is likely to induce disulfidptosis. Moreover, evidence indicates that moderate levels of SLC7A11 in cancer cells may be beneficial, and high levels may suppress tumor metastasis. This may be because metastatic cancer cells with elevated SLC7A11 levels show increased sensitivity to oxidative stress than primary tumor cells [45]. These findings, to some extent, explain the paradoxical question of why high SLC7A11 expression typically promotes cancer development yet induces disulfidptosis.

Nrf2 has also emerged as a key focus in studies on ferroptosis due to its role as an upstream regulator of SLC7A11. The findings from studies on ferroptosis are equally significant in the context of disulfidptosis. Nrf2, a leucine zipper protein, is covalently inhibited via ubiquitination by the regulatory factor Keap1 in the cytoplasm [163, 166]. When cells experience oxidative stress or other pressures, Nrf2 dissociates from Keap1. Then, it enters the nucleus and binds to the antioxidant response element (ARE) within the promoters of its target genes, thereby initiating various responses [167]. Interestingly, DEM can induce the dissociation of the Nrf2-Keap1 complex [163]. In summary, NRF2 plays a crucial role in cellular defense against oxidative stress. Plasma factors in young mice activate Nrf2 expression and reduce oxidative stress in the circulation of aged mice through the Nrf2/Bach1 axis [168]. Some researchers have concluded that the overactivation of Nrf2 induces SLC7A11 expression by directly binding to its promoter region [169]. Accordingly, the role of Nrf2 in promoting SLC7A11 expression has been studied in detail. It has been reported that EBV infection activates the p62-Keap1-Nrf2 pathway and upregulates SLC7A11 in nasopharyngeal carcinoma cells [170]. Another study confirmed that the Keap1/Nrf2 pathway is essential for mitigating oxidative stress in breast cancer cells through system Xc^-^ [167]. Similarly, silencing Nrf2 has been found to significantly reduce SLC7A11 levels, and cells depleted of Nrf2 have exhibited improved vitality during glucose withdrawal. Additionally, there is evidence that Nrf2 can be activated by disrupting mitochondrial thiol homeostasis, in line with the conditions of thiol imbalance in disulfidptosis [171].

The role of Nrf2 in inducing disulfidptosis is an important area of focus for the future. Several studies have reported that Epigallocatechin Gallate (EGCG) can activate Nrf2 and its downstream targets, including SLC7A11 [172, 173]. This is likely due to the enhanced translocation of Nrf2 from the cytoplasm to the nucleus in some cells. There is also evidence that EGCG can inactivate the Keap1 protein to maintain Nrf2 function [174]. Moreover, one study found that treatment with EGCG alone only induces a moderate Nrf2 response, but EGCG treatment under selenium-deficient conditions can produce a strong Nrf2 response [175]. These studies provide vital insights into the effects of EGCG under different conditions.

Drugs other than EGCG have also been found to exert similar effects. For example, tetrathiomolybdate (TTM, a copper chelator) has been found to serve as an Nrf2 activator. TTM treatment promotes the nuclear translocation of Nrf2 and upregulates the transcriptional activity of Nrf2 target genes (including *HMOX1*, *GCLM*, and *SLC7A11*) in human umbilical vein endothelial cells [176]. *Rhodiola rosea* extract can promote the activation of the Nrf2/GPX4 axis and upregulate the protein expression of SLC7A11 and GPX4 [177]. Exogenous H2S can strongly induce the expression of phosphorylated PI3K and AKT as well as the nuclear accumulation of Nrf2 by modulating the PI3K/Akt/Nrf2 pathway. This may further facilitate the role of Nrf2 in promoting SLC7A11 expression [178]. Other drugs, including dexmedetomidine, alginates, iridin, and propofol, may also be effective in this regard [179-182].

Nevertheless, it should be noted that these findings are largely derived from studies exploring whether the SLC7A11 overexpression can inhibit ferroptosis. However, the effects of these drugs on disulfidptosis remain unclear and must be explored further. That said, the current evidence indicates that these drugs can promote SLC7A11 expression to varying degrees. Hence, they likely meet the conditions for inducing disulfidptosis.

#### 6.6.2. Targeting the cystine pool

##### 6.1.2.1. N-acetylcysteine (NAC)

NAC is a therapeutic agent that is widely used to treat acetaminophen overdoses. It is often employed as a direct ROS scavenger (particularly for H_2_O_2_) and as an antioxidant [183, 184]. Notably, its ability to increase the intracellular concentrations of GSH is critical to its application as an antioxidant. NAC acts as a precursor to L-cysteine, expanding the intracellular cysteine pool [185]. However, the mechanism through which it subsequently induces the synthesis of intracellular GSH is not entirely clear. Nevertheless, some evidence suggests that this effect may be mediated by the release of protein thiols via disulfide bond cleavage, thereby increasing GSH levels [186, 187]. Many studies have found that treatment with NAC can attenuate oxidative stress in cells, sometimes promoting tumor proliferation [188]. Our focus lies in understanding whether NAC has any effect on cells undergoing disulfidptosis.

NAC is the most stable form of cysteine during drug delivery. Hence, we speculate that the cysteine produced via NAC hydrolysis may be quickly oxidized to cystine, intensifying intracellular disulfide stress and thus promoting disulfidptosis. However, this raises a key point of contention: how much NAC is oxidized intracellularly, and does the remaining NAC induce GSH elevations and alleviate this stress? Although the answers to these questions are currently unknown, some studies have demonstrated that GSH depletion and GSSG generation during exposure to oxidants are not the cause of actin polymerization (the mechanism of cell death in disulfidptosis). Hence, the variations in GSH content following NAC treatment may not have a decisive effect on disulfidptosis. Moreover, it has been observed that NAC can modulate the levels of cellular free iron via Nrf2, which are responsible for iron-mediated lipid peroxidation, thus inhibiting ferroptosis [188].

Overall, as the current evidence is limited, the effect of NAC in the context of disulfidptosis can only be hypothesized at this stage. Additional research is required to explore the metabolic impact of NAC in disulfidptosis and its association with ferroptosis.

#### 6.6.3. Targeting glucose transport

GLUTs are a group of membrane proteins that transport glucose from the extracellular environment into the cytoplasm. To meet the high energy demands of proliferation, cancer cells often increase their glucose uptake. Current evidence suggests that glucose starvation is an indispensable prerequisite for disulfidptosis. Notably, some GLUT inhibitors have been proven to induce glucose deprivation [10]. This highlights their potential in disulfidptosis-based cancer treatment and underscores the need to develop more GLUT inhibitors.

GLUT1 is known to be upregulated in most types of cancers. Meanwhile, significant GLUT2 elevation has been observed in liver, pancreatic, stomach, and colon cancer. Furthermore, GLUT3 is upregulated in lung, cervical, head, ovarian, breast, and bladder cancer, while GLUT4 is overexpressed in colon, breast, and pancreatic cancer as well as in lymphoma. Table 3 focuses on the inhibition of GLUT via different chemical agents, including novel drugs.

**Table 3:** Potential glucose transport inhibitors.

Potential glucose transport inhibitors or compounds	Target	Mechanism	Ref
**KL-11743**	GLUT1-4	An orally active inhibitor targeting Class I glucose transporters that competes with glucose and effectively inhibits GLUT1-4. This inhibitor selectively blocks glucose metabolism, causing a rapid depletion of NADH stores and inducing the accumulation of aspartic acid.	[189]
**BAY-876**	GLUT1	An orally active selective inhibitor of glucose transporter 1 (GLUT1). It suppresses glycolysis, including stress-regulated glycolysis, in ovarian cancer cells and also hinders their adhesion-dependent and -independent growth. Finally, it inhibits the growth of RB1-positive triple-negative breast cancer.	[190, 191]
**Fasentin**	GLUT-1/4	A glucose uptake inhibitor capable of inhibiting GLUT-1/GLUT-4 transport proteins. It is a known chemical sensitizer and increases the sensitivity of cells to FAS.	[192]
**STF-31**	GLUT1	A selective inhibitor of GLUT1, STF-31 can specifically kill renal cell carcinoma by targeting the glucose uptake mediated by GLUT1.	[193, 194]
**Phloretin**	GLUT1/4	A broad-spectrum inhibitor of various transporters and channels, including GLUTs and aquaporins, phloretin. It induces apoptosis in HepG2 cells by inhibiting the expression of GLUT2. Furthermore, combined treatment with Quercetin and Paclitaxel significantly reduces the growth of HepG2 xenograft tumors in nude mice.	[195]
**Epigallocatechin gallate (EGCG)**	GLUT2/5	It inhibits GLUT2/5 in oocytes from. It inhibits glucose uptake and metabolism in breast cancer cells through an estrogen receptor-independent mechanism.”	[196, 197]
**Hesperidin**	GLUT2/5	It inhibits GLUT2/5 in oocytes from African clawed frogs	[198]
Apigenin	GLUT1-4/7	It inhibits GLUT2 and GLUT7 in Xenopus oocytes and reduces the levels of GLUT1/3 in tumor tissues. Moreover, it ameliorates glucose and lipid metabolic abnormalities in H9c2 cells stimulated by AngⅡ/hypoxia or with overexpression of HIF-1α, through downregulating GLUT-4 expression.	[199]
**Luteolin**	GLUT4	It inhibits GLUT4 in hypertrophic H9c2 cells and Improves abnormal glucose and lipid metabolism induced by angiotensin II/hypoxia through the inhibition of GLUT4	[200]
**Genistein**	GLUT1	It inhibits GLUT1 in human erythrocytes. The combination of celecoxib and genistein induces apoptosis in prostate cancer cells, with the inhibition of Glut-1 playing a key role. The combination of baicalein and genistein delays prostate cancer tumor growth by inhibiting the PI3K/AKT3 signaling pathway and reducing the number of Glut-1 transport proteins.	[201, 202]
**Curcumin**	GLUT1	It directly suppresses basal glucose uptake in L929 fibroblasts.	[203, 204]
**Gossypol**	GLUT1	It inhibits GLUT1 in HL-60 cells and human erythrocytes, and it can downregulate several genes (including GLUT1), hindering the survival of colon cancer cells.	[205]
**SMI277**	GLUT1	It negatively regulates the expression of the GLUT1 protein and induces apoptosis in tumor cells.	[206]
**TZD (F18)**	GLUT1/4	It inhibits GLUT1/4 and can block the cell cycle in the subG0-G1 phase, thus causing cell necrosis and apoptosis.	[207]
**Trehalose**	GLUT1-4/8	It inhibits the activity of glucose transport proteins (GLUT1, GLUT2, GLUT3, GLUT4, and GLUT8) expressed on the cell membrane, thereby inducing the activation and phosphorylation of adenosine monophosphate-activated protein kinase (AMPK).	[208]
Cytochalasin B	GLUT-1	It is a microfilament toxin that can be employed to regulate cellular skeletal elements. Additionally, it serves as a naturally derived GLUT inhibitor.	[209, 210]
**WZB 117**	GLUT1	It is a polyphenol-derived small molecule inhibitor of GLUT1, WZB 117 inhibits glucose uptake in multiple tumor cell lines (A172, BHY, HeLa, HN, HT-29, MG-63)	[194]
**DRB18**	GLUT1-4	It is a synthetic small inhibitory molecule that serves as an effective pan-Class I GLUT inhibitor both *in vitro* and *in vivo* in cancer cells. It can bind to GLUT1-4 in the outward-open conformation, suppressing tumor growth by inhibiting GLUT1-4-mediated glucose transport and metabolism	[211]
**Glutor**	GLUT1-3	It inhibits GLUT1, GLUT2, and GLUT3 in HCT116 cells. Moreover, it may possess an exceptional capacity to overcome the rescue and compensatory mechanisms of highly adaptive tumor cells.	[212]
**Anti-GLUT1 antibody**	GLUT1	It inhibits cell growth and induces apoptosis in breast cancer and non-small cell lung cancer cell lines.	[213]

### 6.7. Immunotherapy

In recent years, immunotherapy has increasingly come into focus, with the blockade of immune checkpoints to enhance the immune response against cancer cells being a major area of interest. For immunotherapy aimed at triple-negative breast cancer (TNBC), a hypoxia-targeted nanomedicine carrying BMS202 (a small molecule antagonist for PD-1/PD-L1 interaction), named BMS202@HZP NPs, has been created. This nanomedicine is used in conjunction with anti-PD-L1 therapy and low-dose radiotherapy (LDRT) [214]. Significantly, this targeted medication depletes NADPH in tumor cells, potentially facilitating disulfidptosis outcomes while also enhancing the efficacy of LDRT. (As NADPH depletion is a crucial initiator of disulfidptosis.) Moreover, it has been discovered that eliminating or inhibiting the gene for 6-phosphogluconate dehydrogenase (6PGD) in the PPP pathway results in the production of high-quality CD8+ T effector cells [215]. This makes 6PGD a likely target for improving tumor immunotherapy, bridging the gap between T cells and disulfidptosis. Furthermore, in the latest advancements, to break the tumor microenvironment shaped by TNBC (which leads to the weakening of the immune system and increases the risk of metastasis), GOx-IA@HMON@IO (an intelligent nanomedicine) has been explored for cancer treatment. This approach leverages the synergistic effects of disulfidptosis, ferroptosis, and immune suppression reversal, and is noted for its excellent biocompatibility and safety. The specific mechanism involves the release of GOx consuming intracellular glucose, leading to the downregulation of NADPH and accumulation of cystine, and triggering disulfidptosis. Additionally, a decline in cysteine levels, coupled with IA's cascade reactions, results in diminished GPX4 and toxic ROS buildup, triggering ferroptosis; Both processes further result in glycolysis and oxidative phosphorylation dysfunction, reversing tumor hypoxia. This is followed by an enhancement in the function of cytotoxic T lymphocytes (CTL). Moreover, an increase in IFNα secreted by a large number of CD8+ T cells is observed. This mechanism further facilitates ferroptosis by suppressing SLC7A11. This discovery not only integrates disulfidptosis with immunity but also mediates the synergistic action between disulfidptosis and ferroptosis, marking a breakthrough [216]. Despite this, more intermediate processes and details about disulfidptosis and immunotherapy need to be proven. Given the metabolic characteristics of disulfidptosis, combining immunotherapy with radiotherapy or chemotherapy could be promising.

### 6.8. Radiotherapy

The traditional mechanism of radiotherapy mainly involves using high-energy radiation to damage the DNA of cancer cells, thereby inhibiting or killing the cancer cells. Recent research has also revealed that radiotherapy directly facilitates cell ferroptosis, expanding the concept of radiotherapy. Nonetheless, due to various factors like cancer cell resistance, enhancing cells' radiosensitivity continues to be a persistent topic. Phy@PLGdH nanosheets, created by integrating emodin methyl ether (Phy) with layered gadolinium hydroxide (PLGdH) nanosheets, can disrupt intracellular NADPH and nucleotide homeostasis, ultimately sensitizing to radiotherapy (RT) and eliciting robust CD8+-T cell-dependent anti-tumor immunity [217]. Another designed antitumor drug, NRA@DH Gel, similarly inhibits the pentose phosphate pathway (PPP) and reduces NADPH production. Its importance lies in the combined effect of chemotherapy and radiotherapy to inhibit tumor cell onset, development, proliferation, and migration [218]. In malignant glioblastoma (GBM) cells, overexpression of SLC7A11 leads to heightened double-strand break levels and increased radiosensitivity. Inhibiting isocitrate dehydrogenase (IDH1) will lower the levels of NADPH, deoxynucleotides, and glutathione in the cells, heightening their sensitivity to radiation-induced aging [219]. Although factors promoting disulfidptosis exist in the above process, it is yet unknown whether disulfidptosis can participate in the sensitization effect of radiotherapy, as disulfidptosis has not yet received widespread attention in this regard. Regardless, this presents a promising prospect.

### 6.9. Chemotherapy

Chemotherapy, a broadly utilized and mature cancer treatment approach, likewise presents potential for application in disulfidptosis. Reports indicate that glioblastomas with IDH1 (R132) mutations experience hindered NADPH production. The reason for prolonged survival in patients with mutated glioblastomas is likely due to the sensitivity of these tumors to radiotherapy and chemotherapy, facilitated by low NADPH levels [220]. In another study, targeting ITPKB resulted in reduced NOX4 activity, redox imbalance, and increased sensitivity of cancer cells to platinum-based therapy. This occurs because IP4, a product of ITPBK, competes with the NOX4 cofactor NADPH for binding [221]. All these factors may be associated with the redox imbalance involved in disulfidptosis. However, it must be acknowledged that current research on the application of chemotherapy in disulfidptosis is limited, and more evidence is needed to demonstrate their connection.

## 7. Challenges

As discussed above, disulfidptosis offers certain possibilities for the treatment of cancer. However, since the research is still in its early stages, there are many challenges and unknowns that need to be explored. Understanding these potential limitations is crucial for the subsequent application of disulfidptosis in cancer treatment.

### 7.1. Drug resistance

#### 7.1.1. Metabolic reprogramming related to NADPH

To combat oxidative stress, cancer cells have evolved a network of signaling pathways and enzyme regulations to maintain NADPH homeostasis. Among these, the Warburg effect (the phenomenon where most tumor cells rely on aerobic glycolysis) is the best known, and its various mechanisms and roles in cancer have been widely reported [222, 223]. Such metabolic reprogramming often leads to different levels of resistance in cancer cells, impacting the efficacy of treatments. Likewise, in disulfidptosis, maintaining NADPH homeostasis greatly counters this form of cell death. Therefore, in this section, we focus on the potential mechanisms of NADPH-related resistance developed by cancer cells to counteract disulfidptosis.

In cancer cells, aside from the PPP pathway, the primary source of NADPH is through folate-mediated one-carbon metabolism. It is reported to support various physiological processes, including biosynthesis, amino acid homeostasis (glycine, serine), etc. Importantly, several processes involve NADPH production: MTHFD1 in the cytoplasm and MTHFD2/L in the mitochondria catalyze the oxidation of 5,10-methylene-THF (CH2-THF) to form 10-formyl-THF, while ALDH1L1 in the cytoplasm and ALDH1L2 in the mitochondria catalyze the oxidation of 10-formyl-THF to CO2 [80]. Studies show that significant increases in MTHFD2 and MTHFD1 are associated with poorer survival rates, and MTHFD1L (another enzyme in the folate cycle) aids in the production and accumulation of NADPH, greatly alleviating oxidative stress in cancer cells. Silencing MTHFD1L can increase oxidative stress, making cancer cells sensitive to sorafenib (a targeted therapy for HCC) [224]. Besides, other factors (including malic enzyme, glutamine metabolism, fatty acid oxidation) are also directly or indirectly involved in the generation and maintenance of NADPH homeostasis. Reports indicate that isocitrate dehydrogenase 2 (IDH2) can stimulate aerobic glycolysis and the PPP bypass in gemcitabine-resistant UC cells. Inhibiting its expression can restore chemotherapy sensitivity. Thus, these processes are closely linked to the generation of NADPH and may serve as mechanisms through which cancer cells counteract NADPH depletion. The enzymes involved may potentially become targets for disulfidptosis in therapeutic applications.

In addition to the previously described metabolic pathways, cancer cells combat NADPH depletion through various other mechanisms. Telomerase reverse transcriptase (TERT) increases NADPH and GSH levels by upregulating glucose transporter GLUT1 and glucose-6-phosphate dehydrogenase (the rate-limiting enzyme of the PPP pathway) [225]. MAPK14 is phosphorylated during nutrient scarcity, increasing the expression levels of SLC2A3 and thereby the uptake of glucose into the cell [226]. Concurrently, it facilitates the metabolic switch from glycolysis to the PPP by controlling the degradation of PFKFB3. As a result, cancer cells manage to survive and build resistance against environments lacking in nutrients. ACSL3 mediates the epithelial-mesenchymal transition (EMT) and metastasis of CRC cells by generating NADPH through activating the FAO pathway [227]. Moreover, positive regulators of G6PD (such as Transcription Factor YY1, NSD2, PBX3, NeuroD1) have been shown to increase pentose phosphate activity, thereby fostering NADPH production [228-231]. Clearly, cancer cells counteract NADPH depletion via a variety of mechanisms, highlighting the complex challenges in leveraging disulfidptosis for therapeutic purposes.

#### 7.1.2. Drug resistance from SLC7A11

Although overexpression of SLC7A11 promotes disulfidptosis, traditionally, its overexpression has been linked to cancer progression and associated with drug resistance. Salubrinal induces resistance to cisplatin in gastric cancer cells by upregulating SLC7A11 [232]. CD44v interacts with SLC7A11 and stabilizes its expression on the plasma membrane, inducing resistance to 5-fluorouracil in gastric cancer. Elevated SLC7A11 expression endows BRAFV600E mutant melanomas with resistance to BRAF and MEK inhibitors. In contrast, suppressing its expression can notably lead to tumor regression [233]. Further consequences of SLC7A11 overexpression entail resistance to geldanamycin in lung cancer, temozolomide in glioblastoma, and gemcitabine in pancreatic cancer [47,234, 235]. Moreover, studies have shown that increased Nrf2 transcriptionally upregulates SLC7A11 and counteracts the accumulation of ROS post-radiation, thereby contributing to radiation resistance in part [236]. Focusing further downstream of Nrf2, NQO1 has been reported to be overexpressed in cisplatin-resistant ovarian cancer cells A2780/CDDP [237]. Another study shows that MAM induces ferroptosis in drug-resistant NSCLC cells by targeting NQO1 [238]. Therefore, the activation of NQO1 may counteract the drug resistance or radioresistance brought by NRF2. Yet, this is not definitive and necessitates additional investigation. Overall,these discoveries provide a distinctive viewpoint on the function of SLC7A11 across different diseases, emphasizing the need to consider the impacts of mentioned drug resistance or radiation resistance when aiming to implement disulfidptosis in certain cancers.

### 7.2. Disulfidptosis and Cell Migration

We have observed that many genes associated with disulfidptosis are related to cell migration or the regulation of the cytoskeleton. This connection is problematic for cancer therapy, as it enables the possibility of cancer cell metastasis and even resistance to drugs. For instance, higher expression of RPN1 is associated with worse clinical characteristics and poorer prognosis. Knocking out RPN1 can inhibit the proliferation and invasion of breast cancer cells in vitro and induce apoptosis triggered by endoplasmic reticulum stress [239]. ACTN4 facilitates the migration of a wide range of cancer cells, leading to adverse outcomes, including colorectal cancer, intrahepatic cholangio-carcinoma, lung cancer, glioblastoma, pancreatic cancer, cervical cancer, melanoma, prostate cancer, gastric cancer, and ovarian cancer. Suppressing or targeting ACTN4 significantly hinders cancer cell development, acting therapeutically. Additionally, ACTN4 is involved in the radiation resistance of cancer cells and resistance to certain drugs [128,240]. IQGAP promotes cancer development and regulates the cancer process and chemotherapy resistance through multiple pathways [241]. We will not list more genes. It's clear that most genes associated with disulfidptosis are, to some extent, classified as oncogenes, which contradicts our desire to induce cell death by promoting the expression of these genes. The migratory characteristics marked by lamellipodia in cells undergoing disulfidptosis have been confirmed, and these genes undoubtedly play some role in this process. The critical task now is to ascertain the specific roles these genes play in disulfidptosis, the effects they generate, and their interrelations. Furthermore, determining whether the expression of these genes is a cause or an effect of disulfidptosis is of great importance. The answers to these questions will illuminate the path for the future application of disulfidptosis in cancer therapy.

### 7.3. The contradiction between disulfidptosis and ferroptosis in therapy

As noted earlier, the regulation of SLC7A11 expression represents a contradiction between disulfidptosis and ferroptosis. This is particularly highlighted in the treatment of cancer. The mechanisms of certain anticancer drugs aimed at ferroptosis (such as Sorafenib, Sulfasalazine, Cyst(e)inase) involve inhibiting SLC7A11 activity or impeding cystine uptake. This is unfavorable for disulfidptosis, or it can be said that these drugs are difficult to apply to disulfidptosis. This evidence appears to present a choice: ferroptosis and disulfidptosis cannot coexist. However, from another perspective, this also presents certain opportunities. Researchers have discovered the SOX2-SLC7A11 regulatory axis in cancer stem-like cells (CSLCs) of lung cancer. Stem cell transcription factor SOX2 endows cancer cells with resistance to ferroptosis by activating SLC7A11. In colorectal cancer (CRC), the expression of SLC7A11 is upregulated, irrespective of whether ferroptosis is induced. In gastric cancer (GC), ferroptosis inducers (such as erastin, sulfasalazine, sorafenib) have been proven to reduce GC cell growth, but resistance to these drugs has already emerged in cancer cells. Additionally, NFE2L2 induces SLC7A11 expression in endometrial cancer (EC), resisting ferroptosis and leading to radioresistance. Therefore, for cancer cells that overexpress SLC7A11 and can evade ferroptosis, aside from inhibiting relevant pathways, we might shift the perspective from ferroptosis to disulfidptosis, as such cells are likely sensitive to nutritional deficiencies (such as glucose starvation) [242, 243]. In a novel study, LGOd1 was found to induce copper death in HCC cells. Specifically, LGOd1's effect disrupts the copper homeostasis and regulates the biological utilization levels of copper, rather than acting as a copper ion carrier or causing an increase in copper content. This discovery provides another perspective: promoting the accumulation of cystine and other disulfides within cells through alternative methods (without affecting or even inhibiting SLC7A11) [244]. Of course, this is just a hypothesis, as based on the current understanding, the high expression of SLC7A11 remains an essential factor for inducing disulfidptosis.

### 7.4. Other potential limitations

Since the metabolic pathways related to NADPH are shared with normal cells, targeting NADPH without affecting normal cells is very difficult to achieve, and how to minimize the toxic effects on non-cancerous cells is a concern that needs to be addressed. Additionally, hyperthermia, as a promising anticancer method, triggers certain cellular responses, such as increased expression of GLUT1 and activity of glucose 6-phosphate dehydrogenase (G6PD) [245]. This is also unfavorable for disulfidptosis.

## 8. Conclusion & Perspective

In conclusion, new inroads in disease research have been made due to novel insights into the mechanisms of disulfidptosis. These findings hold significant implications from the perspectives of disease onset, progression, and treatment. Based on current studies, it can be ascertained that high expression of SLC7A11 (which is commonly observed in tumor cells) and glucose starvation together mediate disulfidptosis (Depleted NADPH triggers disulfide stress, leading to the collapse of the cytoskeleton and ultimately cell death). These two characteristics distinctly differentiate disulfidptosis from other conventional forms of programmed cell death. Moreover, the emergence of lamellipodia is crucial for recognizing disulfidptosis (though the causes of lamellipodia formation remain unknown). Research has shown that genes associated with disulfidptosis are abnormally expressed across 34 tumor types, particularly in multiple myeloma (IGG) and glioblastoma (GBM) [246]. Furthermore, SLC7A11 shows high expression in multiple tumors that affect the excretory system [247]. The intracellular content of NADPH varies significantly between tissues and cell types. For example, the total NADPH levels in the rat liver are about 420 nmol/g wet weight, while those in skeletal muscle are about 30 nmol/g wet weight. Meanwhile, the concentration of NADPH in the cytoplasm of HeLa cells is 3.1 ± 0.3µM [80]. These insights contribute to the understanding of disulfidptosis-related disease pathophysiology. Additionally, disulfidptosis is characterized by unique features such as disulfide stress and the collapse of the actin cytoskeleton, indicating the theoretical feasibility of manipulating this form of cell death. However, research on disulfidptosis is still in its nascent stages. Exploring the mechanistic underpinnings of disulfidptosis and its contribution to various diseases is of crucial theoretical significance and practical value, as this can guide the development of effective and highly targeted treatment strategies. Here, we summarize the issues that may need attention in future research: (1) How does the collapse of the actin network induce final cell death, and what are the specific mechanisms involved? (2) Are there other pathways beyond the Rac-WRC-Arp2/3 signaling axis that mediate disulfidptosis? (3) Is the high expression of SLC7A11 a necessary condition? Can promoting the accumulation of cystine and other disulfides achieve the same effect? (4) It is necessary to further understand the metabolic regulation and coupling related to NADPH in disulfidptosis. This is very important for treatments targeting NADPH. (5) Further exploration of the association between disulfidptosis and ferroptosis, especially involving metabolic pathways related to SLC7A11 and oxidative stress. (6) Explore the relationship between disulfidptosis and cell migration. Seeking therapies that drive cancer cells towards disulfidptosis without the risk of metastasis holds promise. (7) How to integrate disulfidptosis with existing cancer treatment methods (chemotherapy, radiotherapy, immunotherapy) is a vast field. (8) Further clarification of the expression differences of SLC7A11 and GLUT in different cancer cells is helpful. (9) Regarding GLUT inhibitors, developing high-selectivity or isomer-specific inhibitors will reduce side effects. Overall, this review details the mechanisms of disulfidptosis, highlighting significant metabolic features such as NADPH, Cystine, and F-actin. Based on this, we summarize the genes related to disulfidptosis and propose the possibility of applying disulfidptosis in cancer treatment. Finally, we have explored and provided an outlook on the challenges that disulfidptosis might encounter in future studies. However, additional studies are required to delve into the mechanisms of disulfidptosis and its molecular execution. Such research will provide new perspectives for targeted cancer treatments in the future.
